# LncRNA RBAT1 reduces chemosensitivity of cancer cells to carboplatin/paclitaxel by sponging miR‑27b in endometrial carcinoma

**DOI:** 10.1186/s13048-023-01235-w

**Published:** 2023-07-27

**Authors:** Lingye Fan, Chunyan Wang, Ping Zhan, Yaofang Liu

**Affiliations:** 1 Department of Gynaecology, The Affiliated Hospital of Southwest Medical University, Luzhou City, Sichuan Province 646000 P.R. China; 2 Sichuan Treatment Center for Gynaecologic and Breast Diseases (Gynaecology), Luzhou City, Sichuan Province 646000 P.R. China; 3 Department of Reproductive Technology, The Affiliated Hospital of Southwest Medical University, No.25 Taiping Street, Luzhou City, Sichuan Province 646000 P.R. China

**Keywords:** Endometrial carcinoma, Chemosensitivity, miR-27b, RBAT1

## Abstract

**Background:**

A recent study reported the role of long non-coding RNA (lncRNA) RBAT1 in promoting the development of retinoblastoma and bladder cancer. However, its function in other cancers is unclear. We then studied the role of RBAT1 in endometrial carcinoma (EC).

**Methods:**

The expression of RBAT1 and miR-27b in EC and paired non-tumor samples from advanced EC patients, as well as in plasma samples of EC patients and healthy controls were detected by RT-qPCR. The direct interaction between RBAT1 and miR-27b, and the subcellular location of RBAT1 were determined by RNA-RNA pulldown assay and subcellular fractionation assay, respectively.

**Results:**

EC tissues showed increased expression levels of RBAT1 and decreased expression levels of miR-27b compared to that in non-tumor tissues. Moreover, EC patients showed higher plasma expression levels of RBAT1 and lower plasma expression levels of miR-27b compared to that in the controls. Drug-resistant (DR) patients showed higher expression levels of RBAT1 and lower expression levels of miR-27b in both EC tissues and plasma samples. RBAT1 was detected in both nuclear and cytoplasm and it directly interacted with miR-27b. RBAT1 and miR-27b did not affect the expression of each other. Upregulation of RBAT1 promoted the expression of multidrug-resistant-related protein (P-gp, MRP1, and BCRP). Overexpression of RBAT1 and inhibition of miR-27b promoted cell viability and impeded cell apoptosis and cell cycle arrest at G0-G1 phase, while knockdown of RBAT1 and overexpression of miR-27b inhibited cell viability and induced cell apoptosis and cell cycle arrest at G0-G1 phase. Moreover, miR-27b could abolish RBAT1-induced effects on cell viability, apoptosis and cell cycle.

**Conclusion:**

RBAT1 may reduce the chemosensitivity of EC cells to carboplatin/paclitaxel by sponging miR-27b in EC.

**Supplementary Information:**

The online version contains supplementary material available at 10.1186/s13048-023-01235-w.

## Introduction

Endometrial carcinoma (EC) is a type of solid aggressive tumor that originates from endometrium [1]. The recent cancer statistics reported that EC affects about 28 out of 100,000 women each year, and about five in 100,000 women die of this disease every year [2, 3]. In addition, an increased incidence of EC was observed in recent years [3]. EC patients at early stages can be treated with surgical resection of ovaries, fallopian tubes and uterus, and more than 70% of these patients can survive more than five years [4, 5]. However, tumor metastasis to lymph node and distant sites is common [6]. The 5-year survival rate of EC patients with metastatic tumors is below 20%, mainly due to the lack of effective treatments and the development of drug resistant during chemotherapy [7, 8].

Cancer diagnosed at advanced stages can usually be treated with chemical drugs. For advanced EC, carboplatin and paclitaxel are frequently applied to suppress tumor growth [9, 10]. However, a considerable number of EC patients will develop chemoresistance, leading to failure of treatment [9, 10]. Previous studies have shown that multiple signal pathways are involved in the development of chemoresistance in EC cells [11, 12]. Therefore, certain pathways could be potentially targeted to increase chemosensitivity. Long non-coding RNAs (lncRNAs) have been found to be involved in a variety of physiological and pathological processes, especially in cancers [13]. A recent study reported the role of lncRNA RBAT1 in promoting the development of retinoblastoma and bladder cancer [14]. However, its function in other cancers is unclear. We predicted that RBAT1 could interact with microRNA (miR)-27b, which is a critical player in chemoresistance [15]. We then studied the role of RBAT1 in EC, with a focus on its interaction with miR-27b.

## Materials and methods

### Participants and samples

The present study enrolled advanced (Stage III or IV) EC patients (*n* = 48, mean age 50.8 ± 7.6 years old) and healthy controls (*n* = 48, mean age 50.6 ± 7.8 years old) at the Affiliated Hospital of Southwest Medical University between May 2018 and May 2020. This study was approved by the Ethics Committee of this hospital (approval number SM-2017–19). Inclusion criteria were: 1) EC patients were diagnosed for the first time; 2) EC patients without initiated therapy for any clinical disorders within 100 days prior to admission. Exclusion criteria were: 1) recurrent EC; 2) patients complicated with other malignancies or other severe diseases, such as heart diseases and severe infections. Tumor (EC) and paired non-tumor tissue samples were collected from all 48 EC patients either through a biopsy or during surgical resection of the primary tumors. Plasma samples were collected from both patients and controls on the day of admission under fasting condition. All participants signed the informed consent. The 48 patients received carboplatin/paclitaxel treatment, while dosages varied across patients. Among these 48 patients, 20 patients showed drug-resistant (DR) and the rest 28 patients showed no drug resistant (Non-DR). No significant difference in menopausal status was observed between DR and Non-DR groups. The clinicopathologic features of all the patients were summarized in Table 1.

**Table 1:** Correlation between RBAT1 expression and clinicopathological features in patients with endometrial carcinoma

Clinicopathological features	Cases	RBAT1 expression		*p*-values
		Low (*n* = 24)	High (*n* = 24)	
Age	23	11	12	0.77
< 55	25	13	12	
≥ 55				
Distant metastasis				0.24
Negative	28	12	16	
Positive	20	12	8	
Lymph node metastasis				0.04
Negative	27	10	17	
Positive	21	14	7	
ER				0.25
Negative	24	10	14	
Positive	24	14	10	
PR				0.77
Negative	23	11	12	
Positive	25	13	12	

### Cells and cell culture

Ishikawa and HEC-1B human EC cell lines (ATCC) were first subjected to mycoplasma contamination test prior to use. Only mycoplasma-free cells were used. Cells were incubated with DMEM (Sigma-Aldrich) containing FBS (10%), penicillin (100 U/ml) and streptomycin (100 mg/ml). Cells were cultured in an incubator with temperature, humidity and CO_2_ set to 37 °C, 95% and 5%, respectively.

### Transient transfection

miR-27b inhibitor and its negative control (NC inhibitor), small interfering RNA (siRNA) targeting RBAT1 (si-RBAT1, 5’-UCCACUAUCUAGACUUCGUAG-3’) and its negative control (si-NC, 5’UUGUACUACACAAAAGUACUG-3’), were synthesized by GenePharma (Shanghai, China). Full-length sequence of RBAT1 was constructed into a pcDNA3.1 vector to generate pcDNA3.1-RBAT1 (RBAT1) overexpression plasmid. All transfections were performed using Lipofectamine® 2000 (Invitrogen; Thermo Fisher Scientific, Inc.). The cells were harvested at 48 h post-transfection for use in subsequent experiments.

### RNA extraction

Samples were subjected to RNA extraction using GenElute™ Total RNA Purification Kit (Sigma-Aldirch). Samples were first incubated with Buffer RL to achieve cell lysis. Lysates were then centrifuged at 3, 000 g for 10 min, and the supernatant was transferred to RNase-free tubes. RNA Maxi Spin Columns were then used to achieve RNA binding, followed by addition of the Washing buffer A to remove contaminants. Finally, RNA elution was performed with elution buffer A.

### RNA quality analysis, reverse transcriptions (RTs) and qPCR

All RNA samples were subjected to quality and quantity check prior to the subsequent assays. RNA integrity was analyzed using 2100 Bioanalyzer to determine the concentration and RIN value of all RNA samples. All RNA samples showed an RIN value higher than 9.0, indicating high RNA integrity. RNA samples were diluted to about 2, 000 ng per μl with RNase-free water. With 1 μl RNA sample as the template, cDNA samples were prepared by performing RTs using SSRT III (Invitrogen). With cDNA samples as the template (1 μl cDNA in 20 μl system), qPCRs were performed to determine the expression of RBAT1 with 18S rRNA as the internal control. The expression of miR-27b was determined with U6 as the internal control. PCR protocol was: 95 °C for 5 min, then 40 cycles of 95 °C for 10 s and 58 °C for 50 s. Relative expression levels of RBAT1 and miR-27b were calculated by normalization of Ct values to its internal control using the 2-delta delta Ct method. The primers sequences were as following: RBAT1: 5’-ATGUCUAUAGUUGGCUAGCAT-3’ (forward) and 5’-AUGUCAUTCTAUTCUATUUUC-3’ (reverse); miR-27b: 5’-TTTCTCGAGGGATTACCACGCAACCAC-3’ (forward) and 5’-TTTGAATTCGGCTAGCATTCCCAGCAGGAGA-3’ (reverse); 18S rRNA: 5’-GCTTAATTTGACTCAACACGGGA-3’ (forward) and 5’-AGCTATCAATCTGTCAATCCTGTC-3’ (reverse); U6: 5’-CTCGCTTCGGCAGCACA-3’(forward) and 5’-AACGCTTCACGAATTTGCGT-3’ (reverse).

### Analysis of subcellular location

The standard Cell Fractionation Kit (ab109719, Abcam) was used to prepare both nuclear and cytoplasm fractions from 10^7^ cells. Cells were gathered and incubated on ice for at least 10 min, followed by the addition of cell lysis buffer. Cell lysis was achieved in 20 min on ice, followed by the separation of cytoplasm (supernatant) fraction through centrifugation at 1,400 g for 20 min. Cell pellet, which was the nuclear fraction, was further incubated with nuclear lysis buffer. After that, RNAs were extracted, followed by RT-PCR to detect the expression of RBAT1.

### Analysis of RNA-RNA interaction through pull-down

The interaction between RBAT1 and miR-27b was detected by pull-down assay using in vitro transcripts of both RBAT1 and negative control (NC) RNAs labeled with Biotin. The in vitro transcripts were prepared using DuraScribe® T7 Transcription Kit (Lucigen). MEGAclear™ Transcription Clean-Up Kit was then used to purify RNA in vitro transcripts. The labeling of 3’ end was performed using the Pierce™ Biotin 3' End DNA Labeling Kit, followed by RNA purification. The purified RNAs with Biotin labeling were named as Bio-RBAT1 and Bio-NC. Then these two RNAs were transfected into cells using the same method described above. Cells were collected at 48 h post-transfections, followed by cell lysis using cell lysis buffer. Cell lysate was then incubated with streptavidin-agarose beads to pull-down RNA complex. After that, pull-down samples were used for RNA purification. RNA samples were subjected to RT-qPCR to determine the expression of miR-27b.

### Cell viability assays

Cells were harvested after transfections and trypsinized. Cells were then counted and seeded onto a cell plate (96-well, 3,000 cells per well). After that, carboplatin and paclitaxel were added to 25 nM and 2 nM, respectively. Cells were cultivated for another 24 h, followed by addition of MTT (20 μl per well). Cells were cultivated for another 4 h, followed by the addition of DMSO (150 μL). Finally, OD values at 490 nm were measured to reflect cell viability.

### Flow cytometry assay

Flow cytometry was performed to analyze cell cycle distribution and apoptosis. For analysis of cell cycle distribution, Ishikawa and HEC-1B cells were cultured for 48 h and then fixed with 75% ethanol (Sigma), followed by incubation with RNase A and PI solution at 37 °C for 30 min. For apoptosis analysis, after culture for 48 h, the cells were resuspended in binding buffer and then stained with Annexin V-FITC and PI (Beyotime, Shanghai, China) in the dark. The distribution of cells at different phases and apoptosis were analyzed using a flow cytometer. The apoptotic rate of cells was expressed as the percentage of cells stained with Annexin V-FITC positive and PI negative or positive.

### Luciferase reporter assay

Full length cDNA of RBAT1 was cloned into pGL3 plasmids (Promega). Through the aforementioned methods, cells were transfected with RBAT1 + miR-27b or RBAT1 + miRNA NC. Luciferase activity was detected using the Dual Luciferase Reporter Assay Kit (Promega Corporation) on cells harvested at 24 h post-transfection.

### Transwell assays

Cells were transferred to upper Corning transwell chambers containing serum-free medium, while the lower chambers were filled with medium containing 20% FBS. Matrigel-coated membranes were used in invasion assay, while uncoated membranes were used in migration assay. Cells were cultivated for 24 h and then membranes were fixed with paraformaldehyde (4%). After staining with crystal violet solution (0.2%), membranes were washed with PBS and observed under a microscope.

### Statistical analysis

SPSS (version 17.0) was applied to compare datasets and plot images. Student’s *t*-test was used to explore differences between two groups. *P* < 0.05 was statistically significant.

## Results

### The expression of RBAT1 in tissue and plasma samples

**Fig. 1:**
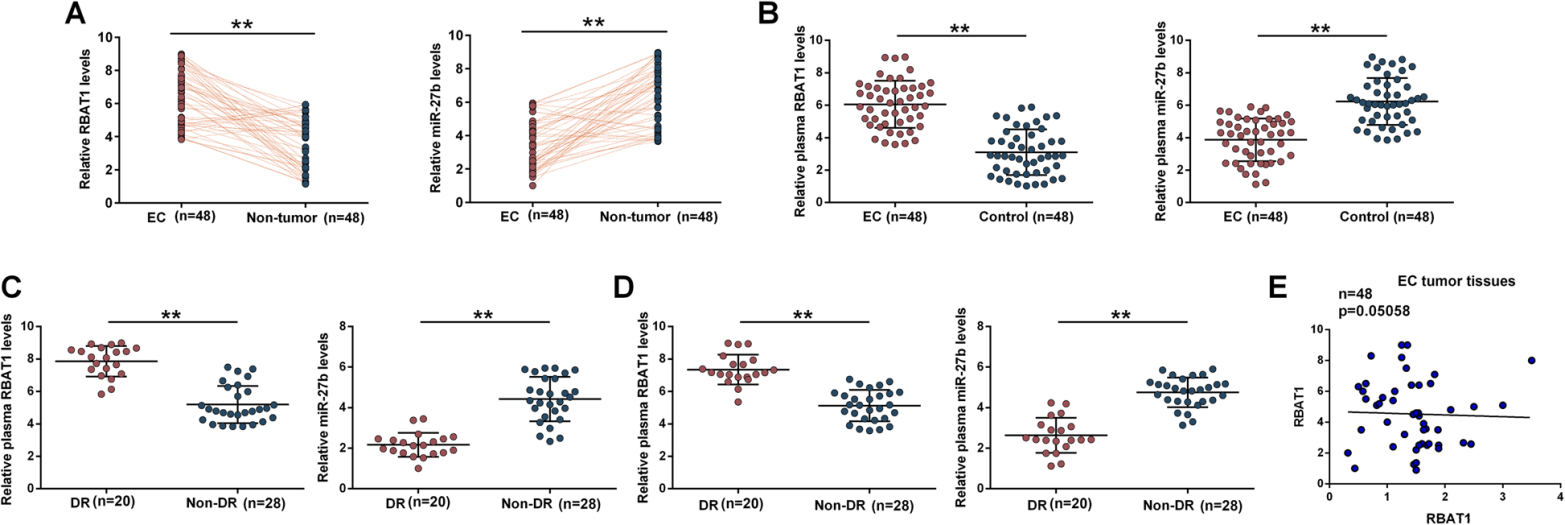
Analysis of RBAT1 expression in tissue samples and plasma. RBAT1 and miR-27b expression in paired EC and non-tumor samples from advanced EC patients (**A**) and plasma samples (**B**) from EC patients and healthy controls was analyzed with RT-qPCR. RBAT1 and miR-27b in EC tissues (**C**) and plasma samples (**D**) were compared between DR and Non-DR groups. **E** Correlations between the expression levels of RBAT1 and miR-27b were analyzed using Pearson’s correlation coefficient. **, *p* < 0.01

The expression of RBAT1 and miR-27b in EC and paired non-tumor tissue samples from advanced EC patients and plasma samples from EC patients and healthy controls were analyzed with RT-qPCR. EC tissues showed increased expression levels of RBAT1 and decreased expression levels of miR-27b compared to that in non-tumor tissues (Fig. 1A, *p* < 0.01). Moreover, EC patients showed higher plasma expression levels of RBAT1 and lower plasma expression levels of miR-27b compared to that in the controls (Fig. 1B, *p* < 0.01). Drug-resistant (DR) patients showed higher expression levels of RBAT1 and lower expression levels of miR-27b in both EC tissues (Fig. 1C, *p* < 0.01) and plasma samples (Fig. 1D, *p* < 0.01). Correlations between the expression levels of RBAT1 and miR-27b were also analyzed and it was observed that RBAT1 and miR-27b were not significantly correlated across EC tumor tissues (Fig. 1E). It is worth noting that RBAT1 and miR-27b were also not closely correlated with each other cross DR (Supplemental Fig. 1A) and Non-DR EC tumor (Supplemental Fig. 1B) tissue samples.

### Subcellular location of RBAT1 and its interaction with miR-27b

**Fig. 2:**
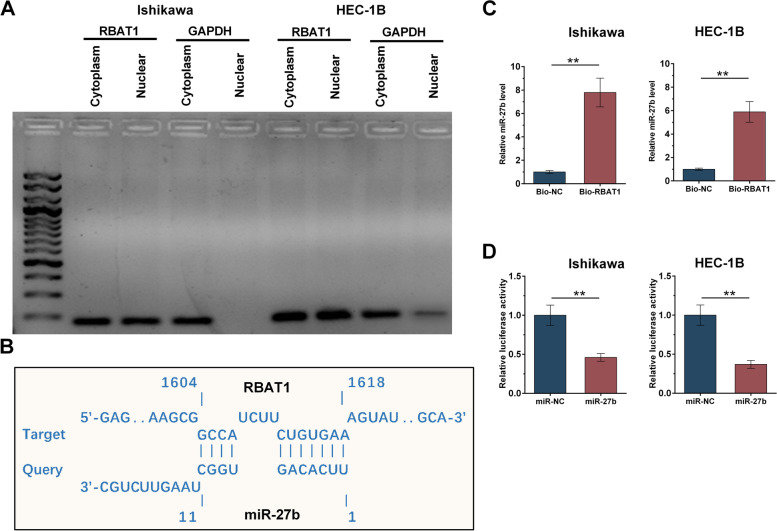
Subcellular location of RBAT1 and its interaction with miR-27b. Both Ishikawa and HEC-1B cells were used to prepare nuclear and cytoplasm fractions, followed by RT-PCR to detect RBAT1 (**A**). IntaRNA 2.0 was applied to predict the direct interaction between them (**B**). RNA-RNA pulldowm assay was applied to determine the direct interaction between them (**C**). Dual luciferase reporter assay was performed by transfecting RBAT1 and miRNA NC or RBAT1 and miR-27b into cells. **, *p* < 0.01

Subcellular location of RBAT1 was detected in both Ishikawa and HEC-1B cells. It showed that RBAT1 was detected in both nuclear and cytoplasm of both cell lines (Fig. 2A). miR-27b has been reported to inhibit cancer progression in a variety of tumors [16]. Therefore, miR-27b was selected to explore its potential role in EC. IntaRNA 2.0 was used to predict the direct interaction between RBAT1 and miR-27b. It was observed that RBAT1 could form multiple base pairings with miR-27b (Fig. 2B). RNA-RNA pulldowm assay showed that the Bio-RBAT1 group had higher expression levels of miR-27b compared to that in the Bio-NC group, confirming the direct interaction between them (Fig. 2C, *p* < 0.01). Dual luciferase reporter assay further confirmed the interaction between RBAT1 and miR-27b (Fig. 2D, *p* < 0.01).

### The role of RBAT1 and miR-27b in regulating the expression of each other

**Fig. 3:**
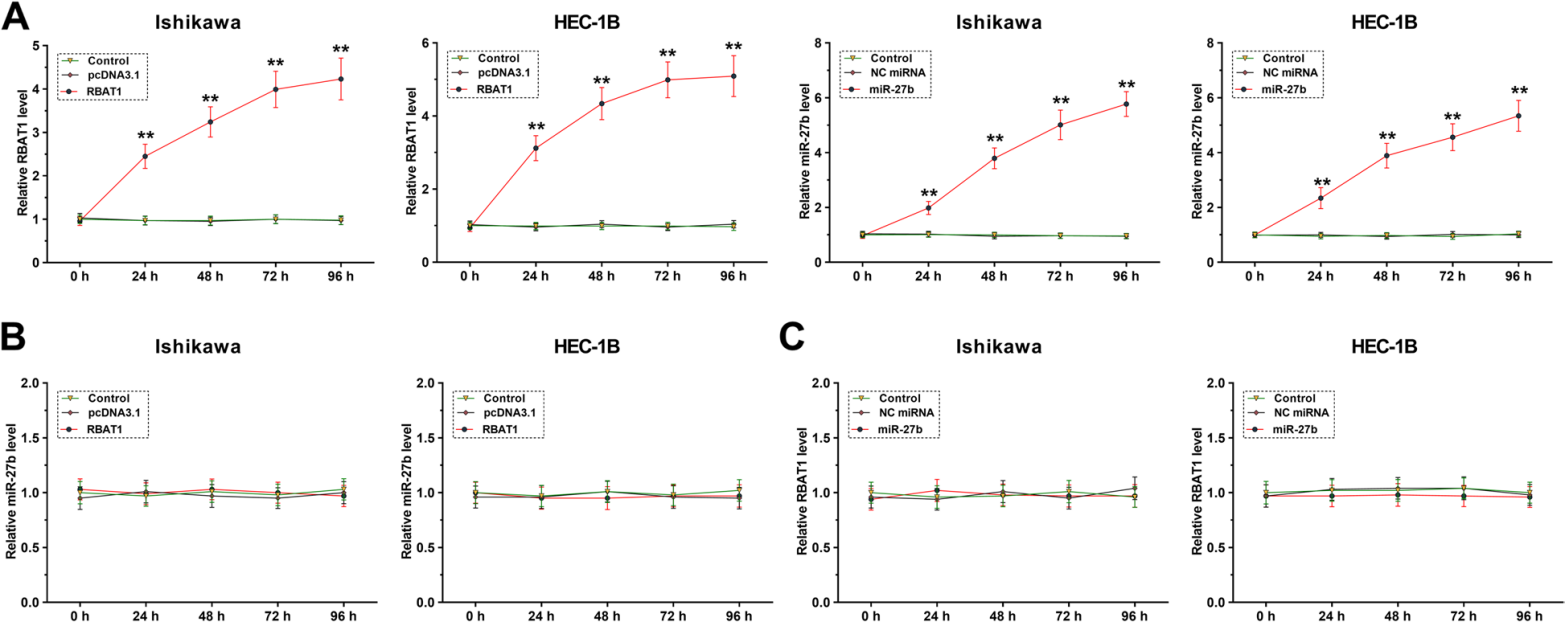
The role of RBAT1 and miR-27b in the expression of each other. Ishikawa and HEC-1B cells were transfected with RBAT1 expression vector or miR-27b mimic to overexpress RBAT1 or miR-27b (**A**). The role of RBAT1 in the expression of miR-27b (**B**) and the role of miR-27b in the expression of RBAT1 (**C**) were analyzed with RT-qPCR. **, *p* < 0.01

RBAT1 or miR-27b was overexpressed in Ishikawa and HEC-1B cells, the overexpression was confirmed every 24 h until 96 h (Fig. 3A, *p* < 0.01). It was observed that overexpression of RBAT1 did not affect the expression of miR-27b (Fig. 3B). Similarly, overexpression of miR-27b also showed no role in regulating the expression of RBAT1 (Fig. 3C).

### The role of RBAT1 and miR-27b in regulating the viability of Ishikawa and HEC-1B cells

**Fig. 4:**
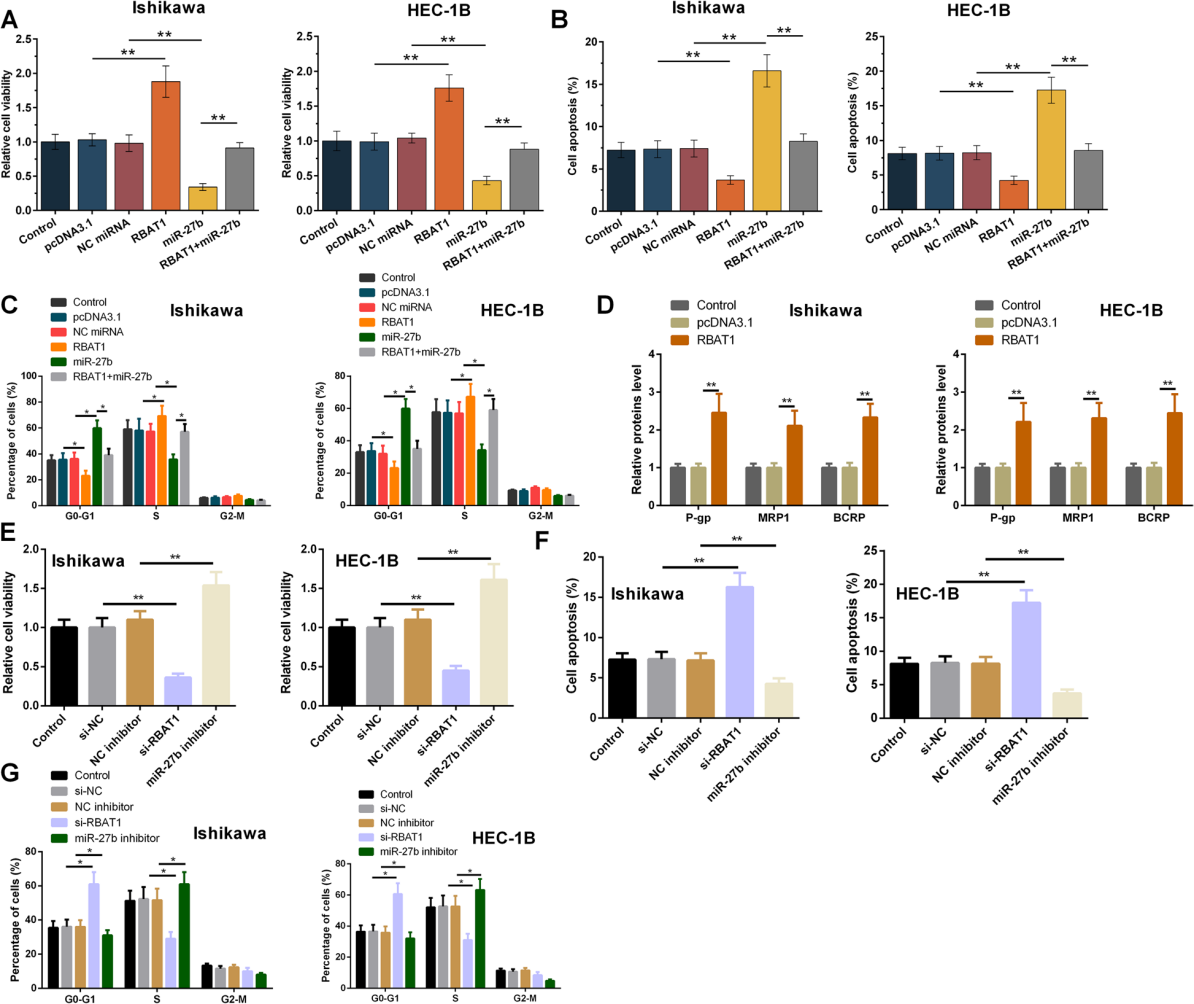
The role of RBAT1 and miR-27b in the viability of Ishikawa and HEC-1B cells. The viability of Ishikawa and HEC-1B cells under carboplatin and paclitaxel treatment was analyzed with MTT assay (**A**). Cell cycle distribution and apoptosis of Ishikawa and HEC-1B cells under carboplatin and paclitaxel treatment were measured by Flow Cytometry (**B** and **C**). Multidrug-resistant-related protein (P-gp, MRP1, and BCRP) expression levels were measured using RT-qPCR in Ishikawa and HEC-1B cells under carboplatin and paclitaxel treatment (**D**). The effect of RBAT1 and miR-27b knockdown on the viability (**E**), apoptosis (**F**), and cycle (**G**) of Ishikawa and HEC-1B cells with carboplatin and paclitaxel treatment. **, *p* < 0.01

The viability of Ishikawa and HEC-1B cells under carboplatin and paclitaxel treatment was analyzed with MTT assay. RBAT1 increased cell viability, while miR-27b decreased cell viability. Moreover, RBAT1 suppressed the role of miR-27b in decreasing cell viability under drug treatment (Fig. 4A, *p* < 0.01). As shown in Fig. 4B, overexpression of RBAT1 inhibited, while overexpression of miR-27b facilitated cell apoptosis, and overexpression of miR-27b counteracted the inhibitory effect of overexpression of RBAT1 on cell apoptosis. As shown in Fig. 4C, overexpression RBAT1 suppressed, while overexpression of miR-27b induced cell cycle arrest at G0-G1 phase. The arrest of cell cycle in Ishikawa and HEC-1B cells caused by overexpression of miR-27b was abrogated by the up-regulation of RBAT1. Furthermore, enhanced expression of RBAT1 accelerated the expression of multidrug-resistant-related protein (P-gp, MRP1, and BCRP) (Fig. 4D, *p* < 0.01). As shown in Fig. 4E, knockdown of RBAT1 inhibited, while silencing of miR-27b promoted cell viability. Knockdown of RBAT1 promoted apoptosis and silencing of miR-27b inhibited apoptosis (Fig. 4F, *p* < 0.01). Moreover, knockdown of RBAT1 induced cell cycle arrest at G0-G1 phase, revealed by increased percentage of cells at G0-G1 phase and reduced distribution at S phase, while knockdown of miR-27b has an opposite effect (Fig. 4G, *p* < 0.01).

## Discussion

This study explored the role of RBAT1 in EC and studied its interaction with miR-27b. We observed altered expression of RBAT1 and miR-27b in EC. In addition, RBAT1 could sponge mature miR-27b in cytoplasm to affect the viability of EC cells during the development of chemoresistance.

In a recent study, it was reported that RBAT1 was upregulated in cell lines and tissue samples of retinoblastoma and bladder cancer [14]. Moreover, RBAT1 may activate E2F3 and interact with HNRNPL to promote tumor progression [14]. To date, the role of RBAT1 in other types of cancer is unclear. In this study we showed that RBAT1 was upregulated in both EC tissues and EC plasma samples. Moreover, EC patients with DR showed higher expression levels of RBAT1 in both EC tissues and plasma samples compared to non-DR patients. Our data suggested that RBAT1 is involved in EC progression and the development of chemoresistance. Our hypothesis was supported by in vitro experiments, in which RBAT1 could increase the viability of two EC cell lines under carboplatin and paclitaxel treatment.

MiR-27b has been characterized as a tumor suppressor in many cancers including EC [17]. For instance, miR-27b is upregulated in EC and its overexpression promotes tumor metastasis by targeting oncogenic MARCH7 [17]. However, the role of miR-27b in chemoresistance EC cells is unclear. However, it is known that miR-27b could increase the sensitivity of cancer cells to chemotherapy in lung cancer cells [15]. In this study we observed that miR-27b was downregulated in EC and its expression was further downregulated by DR. Moreover, overexpression of miR-27b decreased EC cell viability under chemical treatment. Therefore, miR-27b may increase the sensitivity of EC cells to chemotherapy. Moreover, although miR-27b has been reported to regulate cancer cell invasion and migration in EC [17], the present study showed that RBAT1 and miR-27b had no obvious role in regulating the invasion (Supplemental Fig. 2A) and migration (Supplemental Fig. 2B) of Ishikawa and HEC-1B cells. This discrepancy may be explained by different cell lines used in this study. However, our data also suggested that RBAT1 was only closely correlated with lymph node metastasis (*p* = 0.04), but not distant metastasis (*p* = 0.24). The role of RBAT1 in EC metastasis should be further explored.

Interestingly, RBAT1 was detected in both nuclear and cytoplasm and it directly interacted with miR-27b. However, RBAT1 and miR-27b showed no role in regulating the expression of each other. In view of the fact that RBAT1 could suppress the role of miR-27b in decreasing cell viability, we speculated that RBAT1 could sponge miR-27b in cytoplasm to suppress its role in chemoresistance.

**Fig. 5:**
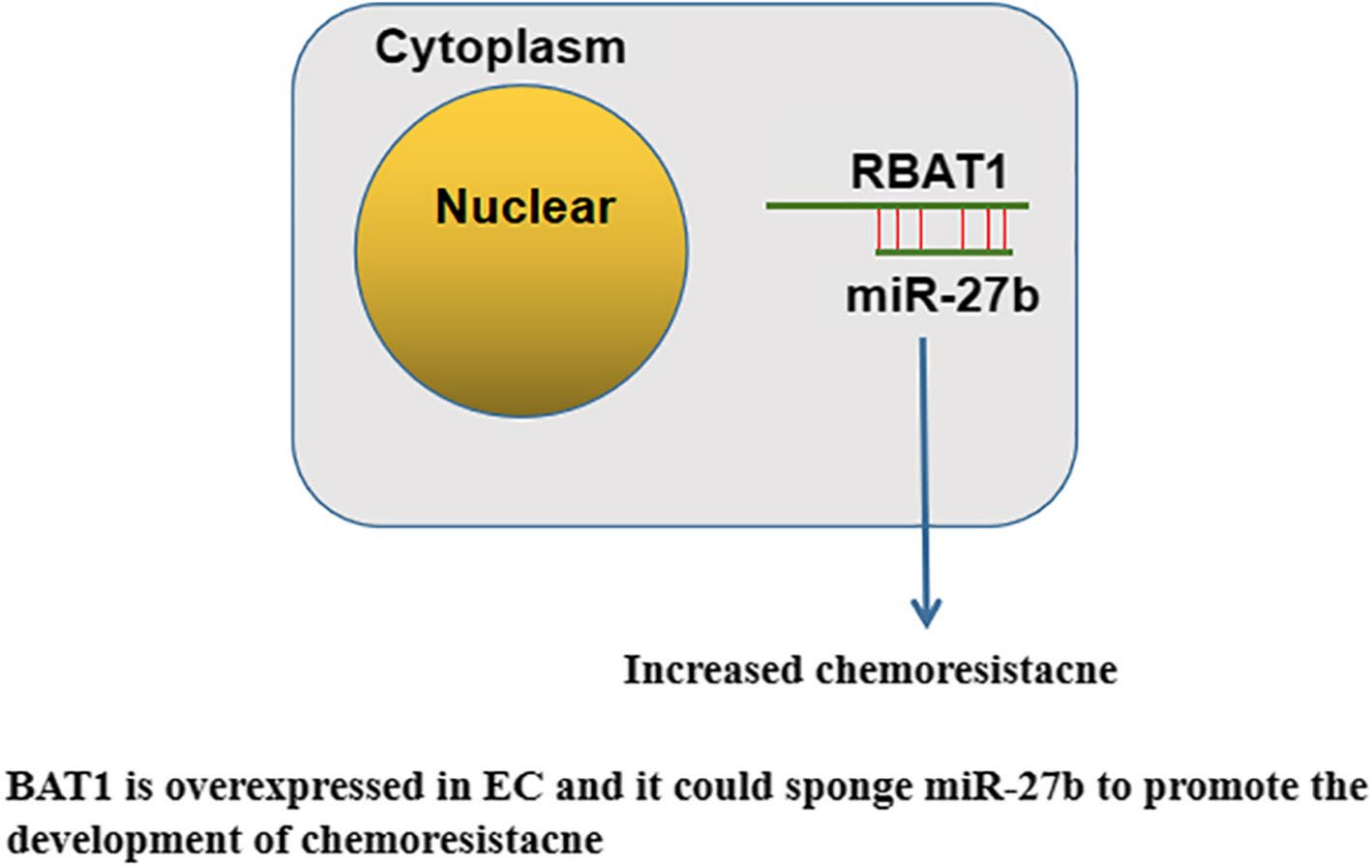
A diagram describing the role of lncRNA RBAT1/miR-27b axis in the development of chemoresistance in EC cells

In conclusion, RBAT1 is upregulated in EC and it could sponge miR-27b to promote the development of chemoresistance (Fig. 5).

## Supplementary Information

**Additional file 1: Supplemental Fig. 1.** Correlations between RBAT1 and miR-27b cross DR (A) and Non-DR EC tumor (B) tissue samples.

**Additional file 2: Supplemental Fig. 2.** The role of RBAT1 and miR-27b in the invasion (A) and migration (B) of Ishikawa and HEC-1B cells.

## Data Availability

The data that support the findings of this study are available on request from the corresponding author.

## References

[1] MacKintosh ML, Crosbie EJ (2018). Prevention Strategies in Endometrial Carcinoma. Curr Oncol Rep.

[2] Felix AS, Yang HP, Bell DW, Sherman ME (2017). Epidemiology of Endometrial Carcinoma: Etiologic Importance of Hormonal and Metabolic Influences. Adv Exper Med Biol.

[3] Constantine GD, Kessler G, Graham S, Goldstein SR (2019). Increased incidence of endometrial cancer following the women’s health initiative: an assessment of risk factors. J Women’s Health (2002).

[4] Bernardini MQ, Gien LT, Lau S, Altman AD, Gilks B, Ferguson SE (2016). Treatment related outcomes in high-risk endometrial carcinoma: Canadian high risk endometrial cancer consortium (CHREC). Gynecol Oncol.

[5] Asher R, Obermair A, Janda M, Gebski V (2018). Disease-free and survival outcomes for total laparoscopic hysterectomy compared with total abdominal hysterectomy in early-stage endometrial carcinoma: a meta-analysis. Int J Gynecol Cancer.

[6] Makker A, Goel MM (2016). Tumor progression, metastasis, and modulators of epithelial-mesenchymal transition in endometrioid endometrial carcinoma: an update. Endocr Relat Cancer.

[7] Farghali MM, Allam IS, Abdelazim IA, El-Kady OS, Rashed AR, Gareer WY (2015). Accuracy of sentinel node in detecting lymph node metastasis in primary endometrial carcinoma. Asian Pac J Cancer Prev.

[8] Brasseur K, Gévry N, Asselin E (2017). Chemoresistance and targeted therapies in ovarian and endometrial cancers. Oncotarget.

[9] Lorusso D, Ferrandina G, Colombo N, Pignata S, Pietragalla A, Sonetto C (2019). Carboplatin-paclitaxel compared to Carboplatin-Paclitaxel-Bevacizumab in advanced or recurrent endometrial cancer: MITO END-2 - a randomized phase II trial. Gynecol Oncol.

[10] Fader AN, Roque DM, Siegel E, Buza N, Hui P, Abdelghany O (2018). Randomized phase II trial of carboplatin-paclitaxel versus carboplatin-paclitaxel-trastuzumab in uterine serous carcinomas that overexpress human epidermal growth factor receptor 2/neu. J Clin Oncol.

[11] Guo F, Zhang H, Jia Z, Cui M, Tian J (2018). Chemoresistance and targeting of growth factors/cytokines signalling pathways: towards the development of effective therapeutic strategy for endometrial cancer. Am J Cancer Res.

[12] Eritja N, Yeramian A, Chen BJ, Llobet-Navas D, Ortega E, Colas E (2017). Endometrial carcinoma: specific targeted pathways. Adv Exper Med Biol.

[13] Prensner JR, Chinnaiyan AM (2011). The emergence of lncRNAs in cancer biology. Cancer Discov.

[14] He X, Chai P, Li F, Zhang L, Zhou C, Yuan X (2020). A novel LncRNA transcript, RBAT1, accelerates tumorigenesis through interacting with HNRNPL and cis-activating E2F3. Mol Cancer.

[15] Zhao R, Wang J, Zhang X, Chen Y (2020). MiR-643 inhibits lipopolysaccharide-induced endometritis progression by targeting TRAF6. Cell Biol Int.

[16] Chen D, Si W, Shen J, Du C, Lou W, Bao C (2018). miR-27b-3p inhibits proliferation and potentially reverses multi-chemoresistance by targeting CBLB/GRB2 in breast cancer cells. Cell Death Dis.

[17] Liu L, Hu J, Yu T, You S, Zhang Y, Hu L (2019). miR-27b-3p/MARCH7 regulates invasion and metastasis of endometrial cancer cells through Snail-mediated pathway. Acta Biochim Biophys Sin.

