# Supplementary figures and images for: LncRNA RBAT1 reduces chemosensitivity of cancer cells to carboplatin/paclitaxel by sponging miR‑27b in endometrial carcinoma
Source: J Ovarian Res. 2023 Jul 27;16:147. doi: 10.1186/s13048-023-01235-w (PMC10375650; doi:10.1186/s13048-023-01235-w)

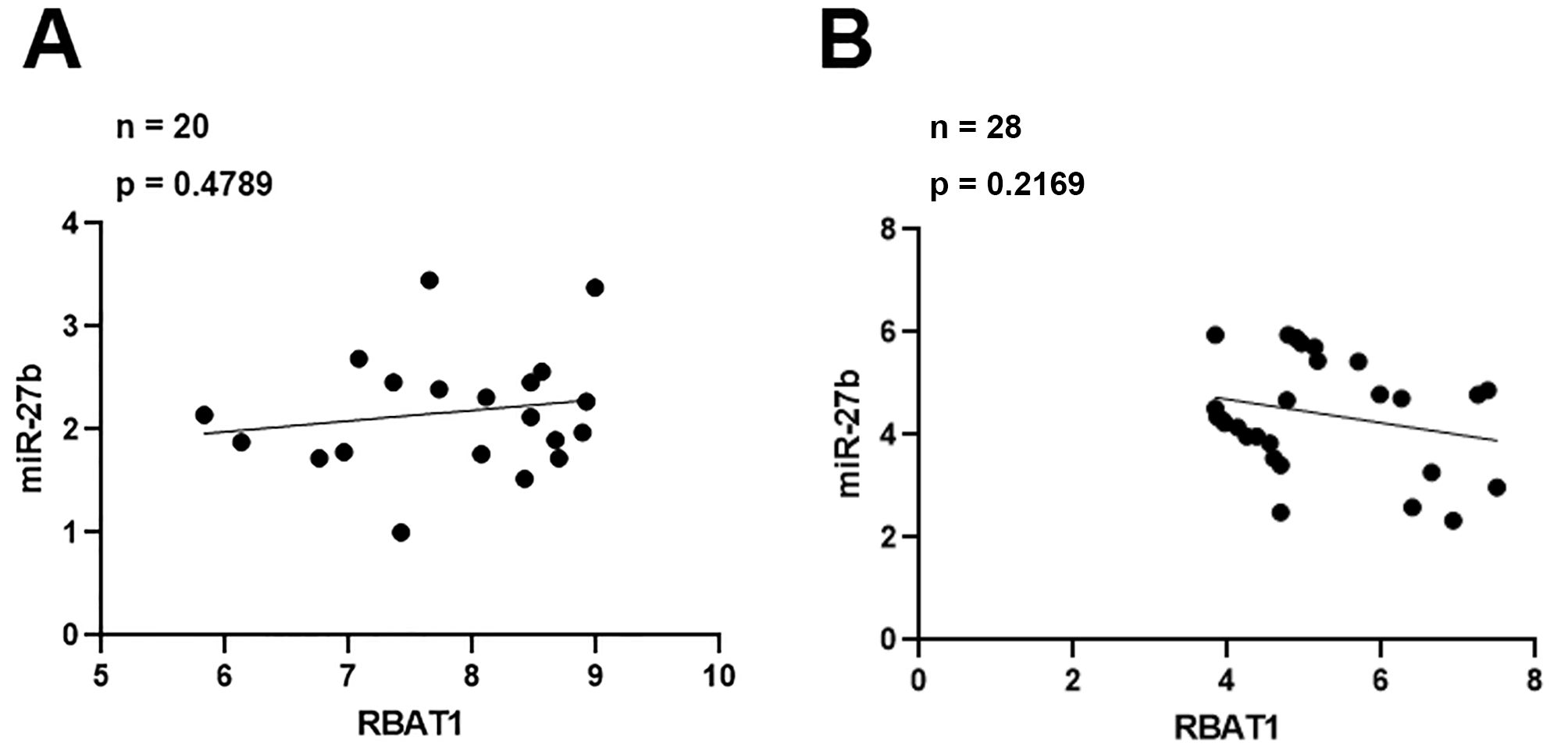

Supplement: Supplementary file 1 — Additional file 1: Supplemental Fig. 1. Correlations between RBAT1 and miR-27b cross DR (A) and Non-DR EC tumor (B) tissue samples. [file 13048_2023_1235_MOESM1_ESM.tif]

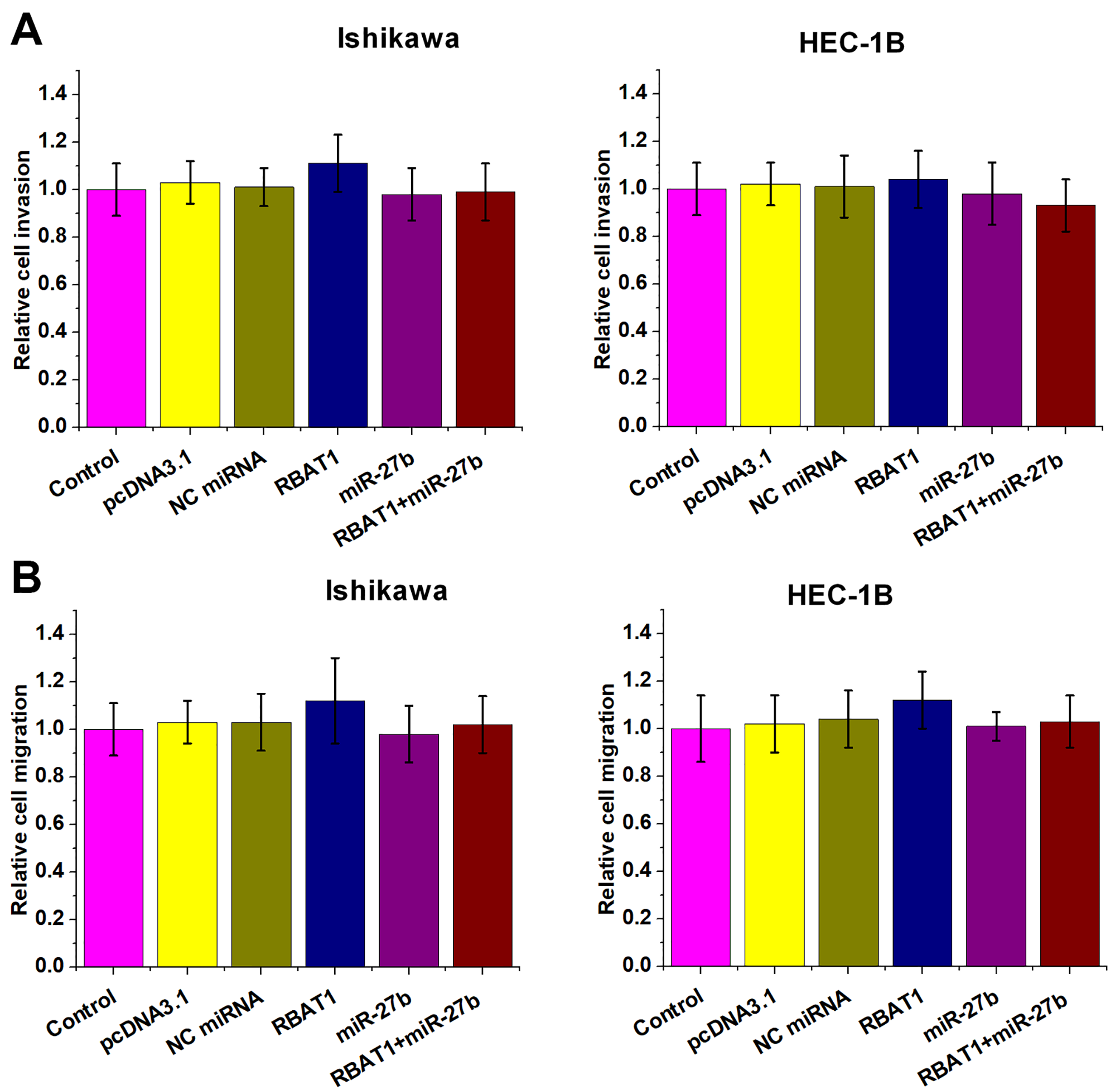

Supplement: Supplementary file 2 — Additional file 2: Supplemental Fig. 2. The role of RBAT1 and miR-27b in the invasion (A) and migration (B) of Ishikawa and HEC-1B cells. [file 13048_2023_1235_MOESM2_ESM.tif]
